# Circulatory resistin levels in inflammatory bowel disease: a systematic review and meta-analysis

**DOI:** 10.1186/s12876-024-03199-7

**Published:** 2024-03-14

**Authors:** Amir Hossein Behnoush, Seyede Parmis Maroufi, Tara Reshadmanesh, Yasmin Mohtasham Kia, Mitra Norouzi, Seyedeh Mina Mohammadi, Aleksandra Klisic, Amirmohammad Khalaji

**Affiliations:** 1 School of Medicine, Tehran University of Medical Sciences, Poursina St., Keshavarz Blvd, 1417613151 Tehran, Iran; 2 Non-Communicable Diseases Research Center, Endocrinology and Metabolism Population Sciences Institute, Tehran University of Medical Sciences, Tehran, Iran; 3 Neurosurgical Research Network, Universal Scientific Education and Research Network, Tehran University of Medical Sciences, Tehran, Iran; 4 Student Research Center, School of Medicine, Zanjan University of Medical Science, Zanjan, Iran; 5 School of Medicine, Iran University of Medical Sciences, Tehran, Iran; 6 Faculty of Life Sciences and Biotechnology, Shahid Beheshti University, Tehran, Iran; 7 Islamic Azad University Tehran Faculty of Medicine, Tehran, Iran; 8 Faculty of Medicine, University of Montenegro, Podgorica, Montenegro; 9 Center for Laboratory Diagnostics, Primary Health Care Center, Podgorica, Montenegro

**Keywords:** Inflammatory Bowel Disease, Ulcerative Colitis, Crohn, Resistin, Systematic Review, Meta-analysis

## Abstract

**Background:**

Inflammatory bowel disease (IBD), including ulcerative colitis (UC) and Crohn’s disease (CD), is a chronic relapsing-remitting systemic disease of the gastrointestinal tract with rising incidence. Studies have shown that adipocytes play a crucial role in patients with IBD by actively participating in systemic immune responses. The present study was designed to investigate the correlation between the circulatory levels of resistin, as an adipokine, and active and remission phases of IBD in comparison with healthy controls.

**Methods:**

Relevant articles were retrieved from PubMed, Embase, the Web of Science, and Scopus from inception until June 2023. Estimation of the standardized mean difference (SMD) and 95% confidence interval (CI) for comparison of plasma/serum resistin levels between IBD patients, patients in remission, and healthy controls were conducted through random-effect meta-analysis.

**Results:**

A total of 19 studies were included, assessing 1836 cases. Meta-analysis indicated that generally, serum/plasma resistin levels were higher in IBD patients in comparison with healthy controls (SMD 1.33, 95% CI 0.58 to 2.08, *p*-value < 0.01). This was true for each of the UC and CD separate analyses, as well. Moreover, it was shown that higher serum/plasma resistin levels were detected in the active phase of IBD than in the remission phase (SMD 1.04, 95% CI 0.65 to 1.42, *p*-value = 0.01). Finally, higher serum/plasma resistin levels were found in the remission phase compared to healthy controls (SMD 0.60, 95% CI 0.15 to 1.06, *p*-value < 0.01).

**Conclusion:**

The results of this systematic review and meta-analysis support the conclusion that circulating resistin levels are increased in IBD (both UC and CD). Also, higher resistin levels were recorded in the remission phase of IBD in comparison with healthy controls. This indicates that further studies may provide valuable insights into the role of resistin in the pathogenesis of IBD.

**Supplementary Information:**

The online version contains supplementary material available at 10.1186/s12876-024-03199-7.

## Introduction

Inflammatory bowel disease (IBD) is a multifactorial disorder characterized by chronic and relapsing inflammation of the gastrointestinal tract [1]. IBD is a lifelong condition affecting 5 million people worldwide and imposes a substantial economic burden on the healthcare system [2, 3]. Since early and effective treatment is crucial for improving patient outcomes in IBD, it is necessary to find a proactive monitoring tool to ensure the efficacy of treatment strategies [4, 5]. Consequently, there is a pressing need to identify more accurate markers that can substantially improve our ability to detect the disease at its early stages, estimate its progression, and facilitate the development of more effective treatment approaches.

Recent studies have revealed that adipose tissue beyond its function in energy storage, acts as a dynamic endocrine organ involved in inflammatory processes. The cellular composition of adipose tissue (including adipocytes, pre-adipocytes, macrophages, and leukocytes) contributes to immunological functions and the release of various inflammatory mediators such as TNF, IL-6, IL-1, and adipokines [6–11]. Adipokines are cytokine mediators released by adipocytes. They have significant roles in regulating various metabolic functions and the immune system. Among these adipokines, leptin, adiponectin, and resistin specifically play critical roles in modulating inflammation [12, 13].

Resistin, a 12.5-kDa cysteine-rich peptide (also known as adipocyte secreted factor or FIZZ-3), exhibits diverse biological effects and has been studied as an inflammatory marker connecting metabolic and inflammatory pathways [14, 15]. While initially recognized as an adipocyte-secreted factor involved in insulin resistance, resistin is predominantly expressed in monocytes, macrophages, spleen, bone marrow-derived cells, and adipose cells, albeit at low levels [16]. The NF-κB signaling pathway activates the expression of resistin in response to pro-inflammatory cytokines including IL-1, IL-6, and TNF-α [17]. Interestingly, resistin promotes the production of TNF-a and IL-12, creating a positive feedback circle. Moreover, resistin plays a notable role in activating Th1 and Th17 cells, which are the main immune cells involved in the pathogenesis of CD [16, 18, 19]. Given the involvement of effector immune cells such as CD4 + and CD8 + cytotoxic T cells, as well as Th17 cells, along with inflammatory cytokines like TNF-α, IL-1, and IL-6 in the pathogenesis of IBD, the regulatory pathways in which resistin is implicated suggest its potential role in IBD, as demonstrated in previous studies [19–21]. Our aim was to elucidate the significance of resistin in the inflammatory process associated with IBD, revealing its utility as a valuable diagnostic marker and monitoring tool, as well as a screening marker for evaluating the effectiveness of therapy. This article provides a comprehensive review of the role of resistin as an inflammatory marker in patients with IBD. Additionally, a meta-analysis was conducted to compare the levels of resistin in the serum/plasma of patients with active disease, those in remission, and healthy controls.

## Methods

This systematic review and meta-analysis was conducted according to the Preferred Reporting Items for Systematic Reviews and Meta‐analysis (PRISMA) statement [22]. This study’s protocol was registered in PROSPERO with registration number CRD42023432991.

### Search strategy

We did a broad systematic search in the online databases, namely PubMed, Embase, Web of Science, and Scopus using the Medical Subject Heading (MeSH) terms and search queries including “inflammatory bowel disease“OR “Crohn’s disease” OR “ulcerative colitis” AND “resistin” OR “adipose tissue-specific secretory factor definition” OR “resistin-like molecule”. Further information and a complete keywords list are all shown in detail in Supplementary Table 1. We selected studies that compared plasma or serum levels of resistin in patients with IBD and healthy controls in the remission phase after treatment and also studies that evaluate resistin as a promising inflammatory biomarker to assess IBD activity in patients.

### Study selection

After removing duplicates using the EndNote® software, two of our reviewers (AHB and AK) did the initial screening and then selected the studies that met the inclusion criteria: [1] studies that measured plasma or serum levels of resistin in IBD and compared them with healthy controls, [2] studies that measured plasma or serum levels of resistin in UC and compared them with healthy controls [3] studies that measured plasma or serum levels of resistin in CD and compared them with healthy controls [4] studies that measured blood level of resistin in the active phase of disease compared with remission phase, and [5] studies that compared resistin level in the active phase of the disease with controls.

Exclusion criteria were: [1] reported resistin levels in other human samples than blood, [2] not reporting the exact resistin levels, [3] review articles, abstracts, case reports, letters, meetings, animal studies and non-English studies.

We also defined the PICO (population, intervention, control, and outcome) as:

 (P): patients with IBD including UC or CD.

 (I): measuring resistin levels in IBD patients, healthy controls, and individuals in the active and remission phases.

 (C): healthy controls, and individuals in the remission phase.

 (O): Could resistin levels be a promising biomarker with significant alteration in patients in the flaring phase of the disease versus healthy controls and those in the active remission phase?

### Data extraction

Studies were evaluated by two authors (AHB and SPM), and the following data were extracted from each eligible article: (1) first author’s name (2) the year of study and the location (3) serum/plasma level of resistin (4) the demographic characteristic of the cases [type of IBD, disease activity, sample size, male percentage, age, body mass index (BMI)] (5) the population under investigation and (6) main findings of each study. Any discrepancies were resolved through discussion with a third author (TR).

### Quality assessment

The quality of the included studies was independently assessed by two reviewers (AHB and AK) using the Newcastle-Ottawa Quality Assessment Scale (NOS) to examine the selection of participants and study design, comparability of groups, ascertainment of exposure, and outcome processing [23]. The three perspectives of bias in NOS (selection, comparability, and outcome) for cohorts and case-control studies were categorized as “very good”, “good”, “satisfactory”, or “unsatisfactory” regarding the scores of 9–10, 7–8, 5–6, and less than 5, respectively.

### Statistical analysis

We used the random effect meta-analysis in this study to calculate the standardized mean difference (SMD) and 95% confidence interval (CI) for comparison between targeted groups, including IBD patients vs. control, CD vs. control, UC vs. control, and active disease vs. remission.

All analyses were performed in R version 4.3.0 (R Core Team [2020]. R: A language and environment for statistical computing. R Foundation for Statistical Computing, Vienna, Austria) with package “metafor” and a *p*-value less than 0.05 was considered statistically significant. In the studies where the intended data were reported as median and interquartile ranges (IQRs), Luo et al. and Wan et al. methods were used to convert data into mean and standard deviation (SD) [24, 25]. Means and SDs were merged in appropriate conditions using the Cochrane Handbook [26]. To assess heterogeneity, we used the Q test and also, the I-square test to quantify the percentage of heterogeneity. Egger’s statistical tests followed by the funnel plot visual assessment were utilized to recognize publication bias [27]. Additionally, sensitivity analysis by the “leave-one-out” method was performed to assess the impact of each individual study on the overall effect size. Finally, univariate metaregression based on the mean age of the population, male percentage, mean BMI, sample size, and publication year was performed to assess the effect of these variables on the overall variance observed in meta-analyses.

## Results

### Literature search and included studies

**Fig. 1:**
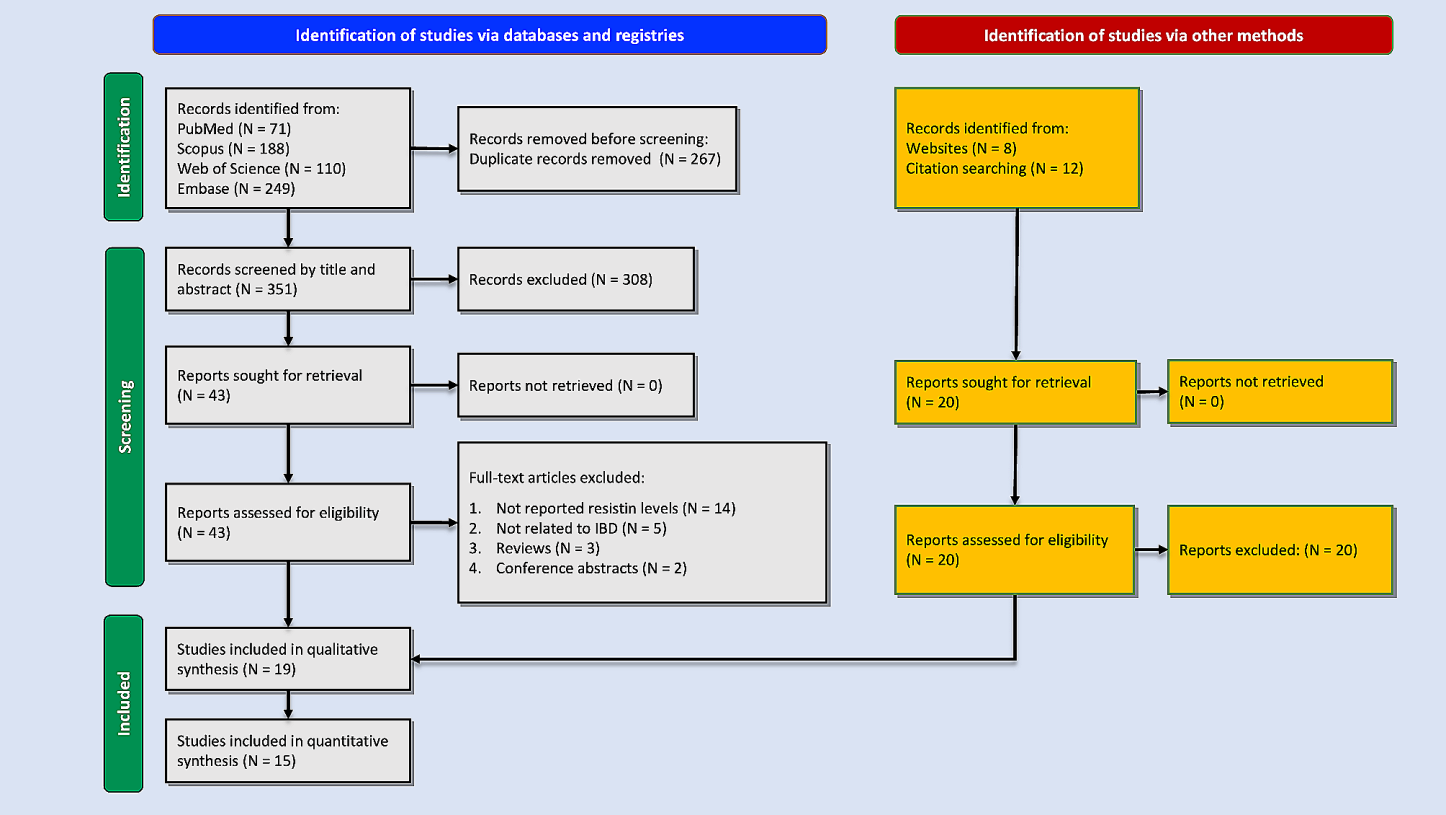
Flow diagram summarizing the selection of studies based on the PRISMA guidelines

The search included 618 results from four databases (PubMed: 71, Scopus: 188, Web of Science: 110, and Embase: 249). After removing 267 duplicates and removing 308 records based on title/abstract screening, 43 records were sought for full-text examination. Finally, 19 studies were included in this review [7, 20, 28–42]. Figure 1 shows the PRISMA flowchart indicating the search, screening, and reasons for exclusion. Studies were conducted between 2006 and 2023 and included 516 patients with UC, 652 patients with CD, and 598 healthy controls. Sixteen studies evaluated resistin levels in serum [7, 20, 28–32, 16–36, 39–42], while three studies used plasma samples [33, 37, 38]. The baseline characteristics and main findings of all included studies are available in Table 1. Quality assessment based on NOS found good and very good qualities in included studies details of which are available in Supplementary Table 2.

**Table 1:** Characteristics of studies evaluating resistin levels in IBD patients

Author	Year	Design	Location	Specimen	Population	IBD type(s)	Sample size	Age	% Male	BMI	Active Disease (%)	Main Findings
**Abdel Kedar et al.**	2010	Cross-Sectional	Egypt	Serum	IBD patients and age and sex-matched HC	UC CD	UC: 24 CD: 16 HC: 20	UC: 32.3 ± 14.3 CD: 29.4 ± 13.6 HC: 30.8 ± 12.0	NR	UC: 24 ± 2.3 CD: 23.7 ± 2.2 HC: 24.2 ± 2.1	UC: 58 CD: 37.5	Patients with IBD had higher levels of resistin compared with HC. Also, active disease was associated with higher resistin levels.
**Abedimanesh et al.**	2018	Case-control	Iran	Serum	UC patients and age- and sex-matched HC	UC	UC: 50 HC: 43	UC: 33.3 ± 9.7 HC: 33.2 ± 8.8	UC: 56 HC: 55.8	UC: 24.8 ± 5.1 HC: 23.1 ± 6.2	NR	Patients with UC had significantly higher levels of resistin, compared with controls (*p*-value = 0.004).
**Bostrom et al.**	2011	Cross-Sectional	Sweden	Serum	IBD patients and HC	UC CD	UC: 53 CD: 51 HC: 40	UC: 45 (23–77) CD: 43 (26–67) HC: 44 (24–67)	UC: 49.1 CD: 54.9 HC: 25	NR	UC: 15 CD: 37	Significantly higher levels of resistin were found in patients with CD compared with patients with NAFLD. Also, patients with hepatobiliary inflammation had higher levels of serum resistin than patients with IBD.
**Frivolt et al.**	2018	Retrospective Case-control	Germany	Serum	CD patients and healthy sex- and BMI-matched controls	CD	CD: 18 HC: 15	CD: 15 ± 1.5 HC: 13.4 ± 1.6	CD: 55.5 HC: 46.7	CD: 18 ± 2.2 HC: 18.4 ± 2.0	UC: 44.4	Before infliximab therapy, patients with CD had significantly higher levels of resistin (*p*-value = 0.002).
**Karaskova et al.**	2022	Case-control	Czech Republic	Serum	IBD patients and sex- and age-matched HC	UC CD	UC: 27 CD: 31 HC: 20	UC: 15 (13–15) CD: 15 (13–16) HC: 15 (12–16)	IBD: 55.2 HC: 55	UC: 20.0 (17.0-22.1) CD: 20.3 (17.3–24.0) HC: 19.1 (16.1–21.5)	NR	Patients with IBD had significantly higher levels of resistin, compared with HC. Also, resistin was higher in each subgroup (CD and UC).
**Karmiris et al.**	2006	Cross-Sectional	Greece	Serum	IBD patients and HC	UC CD	UC: 46 CD: 54 HC: 60	UC: 46 CD: 37 HC: 36	UC: 65.2 CD: 51.8	UC: 25.5 CD: 24	NR	Mean serum resistin levels were significantly higher in both UC and CD patients as compared with HC. There was no difference in adipocytokines in patients with active disease compared with inactive cases (*p*-value > 0.05).
**Karmiris et al.**	2007	Prospective	Greece	Serum	IBD patients	UC CD	UC: 3 CD: 17	UC: 43.3 CD: 37.9	UC: 66.7 CD: 58.8	NR	NR	Serum resistin levels decreased after infliximab therapy in patients with IBD.
**Konrad et al.**	2007	Cross-Sectional	Germany	Plasma	IBD patients and HC	UC CD	UC: 112 CD: 235 HC: 144	UC: 44.6 ± 14.0 CD: 40.4 ± 14.0 HC: 38.2 ± 11.4	UC: 60.7 CD: 46.4 HC: 49.3	UC: 23.9 ± 4.0 CD: 23.4 ± 4.3 HC: 23.7 ± 3.3	UC: 58.9 CD: 56.6	Resistin was higher in both CD and UC patients, compared with HC. Also, in each group, high CRP group and active disease had significantly higher levels of resistin.
**Kurowski et al.**	2021	Prospective	United States	Serum	CD patients and normal colonoscopy HC	CD HC	CD: 36 HC: 68	CD: 13.0 (7.0–18.0) HC: 14.5 (6.0–20.0)	CD: 53 HC: 38	NR	NR	Resistin levels were higher in peditric CD patients compared to HC. These levels were improved significantly after treatment with biologics compared with not on biologics.
**Moreno et al.**	2020	Cross-Sectional	Spain	Serum	CD patients and HC	CD HC	CD: 40 HC: 36	CD: 45.5 ± 12.6 HC: 51.4 ± 15.1	CD: 47.5 HC: 44.4	CD: 25.1 ± 4.1 HC: 24.4 ± 3.9	CD: 45	Resistin levels were different between active CD (aCD), quiescent CD (qCD), and HC groups. The ROC-AUC was 0.59, 0.66, and 0.75 for comparison of qCD with HC, qCD with aCD, and aCD with HC, respectively.
**Morshedzadeh et al.**	2023	RCT	Iran	Serum	UC	UC	UC: 70	UC: 31.2 ± 10.2	UC: 57.8	UC: 23.7 ± 2.5	NR	Flaxseed supplementation led to a significant reduction in resistin levels (-4.85 ± 1.89 vs. -1.10 ± 2.25, *p*-value < 0.001).
**Sobolewska-Włodarczyk et al.**	2020	Prospective cohort	Poland	Serum	IBD patients	UC CD	UC: 35 CD: 30	UC: 42.5 ± 17.8 CD: 38.7 ± 12.5	UC: 51 CD: 53	UC: 21.3 ± 2.1 CD: 20.8 ± 1.9	NR	In patients with IBD, those with poor sleep had significantly higher levels of resistin (*p*-value = 0.0458).
**Theocharidou et al.**	2016	Cross-Sectional	Greece	Plasma	IBD patients and age-, sex-, BMI, and smoking-matched HC.	UC CD	UC: 15 CD: 29 HC: 44	IBD: 36.1 ± 10.3 HC: 37.2 ± 10.7	IBD: 50 HC: 50	IBD: 23.7 (16.5–37.4) HC: 24.3 (18-36.3)	UC: 26.7 CD: 27.6	Resistin was significantly higher in patients with IBD, compared to HC. Also, active disease was associated with higher levels of resistin.
**Titus et al.**	2023	Cross-Sectional	United States	Plasma	IBD patients and HC	UC CD	UC: 10 CD: 5 HC: 7	NR	UC: 40 CD: 80 HC: 57.1	NR	NR	Resistin was significantly higher in patients with IBD (UC and CD). Also, the ROC-AUC was 0.82 and 0.77 for UC and CD, respectively.
**Trejo-Vazquez et al.**	2018	Case-control	Mexico	Serum	IBD patients and HC	UC CD	UC: 23 CD: 11 HC: 19	IBD: 54.8 ± 15.1 HC: 53.2 ± 9.6	IBD: 41.2 HC: 15.8	IBD: 27.6 ± 5.9 HC: 28.4 ± 6.5	NR	Resistin was not different between patients with IBD and HC groups. Also, there was no difference between UC and CD.
**Valentini et al.**	2009	Prospective cohort	Germany	Serum	IBD patients and age- and BMI-matched HC	UC CD	UC: 44 CD: 49 HC: 37	Active UC: 42 (33–52) Quiescent UC: 42 (30–56) Active CD: 32 (26–43 Quiescent CD: 36 (27–45) HC: 39 (30–46)	UC: 34.4 CD: 26.9 HC: 16.2	Active UC: 22.3 (20.2–24.9) Quiescent UC: 24.2 (21–27) Active CD: 20.5 (18.8–24.3) Quiescent CD: 22 (20.3–26.1) HC: 22.3 (20.7–24.2)	UC: 27.9 CD: 26.9	Compared to HC group, patients with active IBD had significantly higher resistin levels, however, patients in remssion did not have different serum resistin in comparison with HC. There was a correlation between resistin and disease activity score (CDAI and CAI). Moreover, it had relation with all inflammatory markers except IL-6.
**Waluga et al.**	2014	Cross-Sectional	Poland	Serum	IBD patients and age- and sex-matched HC	UC CD	UC: 16 CD: 24 HC: 16	UC: 33.2 ± 21.9 CD: 31.0 ± 9.4 HC: 30.3 ± 12.2	UC: 44 CD: 46 HC: 50	UC: 23.4 ± 5.6 CD: 21.4 ± 2.8 HC: 22 ± 4.8	NR	Baseline resistin levels were significantly higher in patients with UC and CD than HC. Treatment to achieving remission resulted in significant decrease in UC patients.
**Youssef et al.**	2022	Cohort	Egypt	Serum	UC patients	UC	UC: 40	UC: 32.7 ± 10.1	UC: 55	NR	NR	Active UC patients had significantly higher resistin levels, in comparison with those in remission.
**Zekri et al.**	2015	Case-control	Egypt	Serum	IBD patients and HC	UC CD	UC: 18 CD: 6 HC: 29	IBD: 41.67 ± 3.2 HC: 43.07 ± 2.79	IBD: 70.8 HC: 59.3	NR	NR	Patients with IBD and HC groups did not show significant difference in terms of resistin levels.

Data are presented as mean ± standard deviation, median [interquartile range], median [range], or percentage. IBD: inflammatory bowel disease, UC: ulcerative colitis, CD: Crohn’s disease, HC: healthy control, BMI: body mass index, AUC: area under the receiver operating characteristic curve, CI: confidence interval, NA: not available, NR: not reported

### Meta-analysis of resistin levels in comparison of patients with IBD and healthy controls

**Fig. 2:**
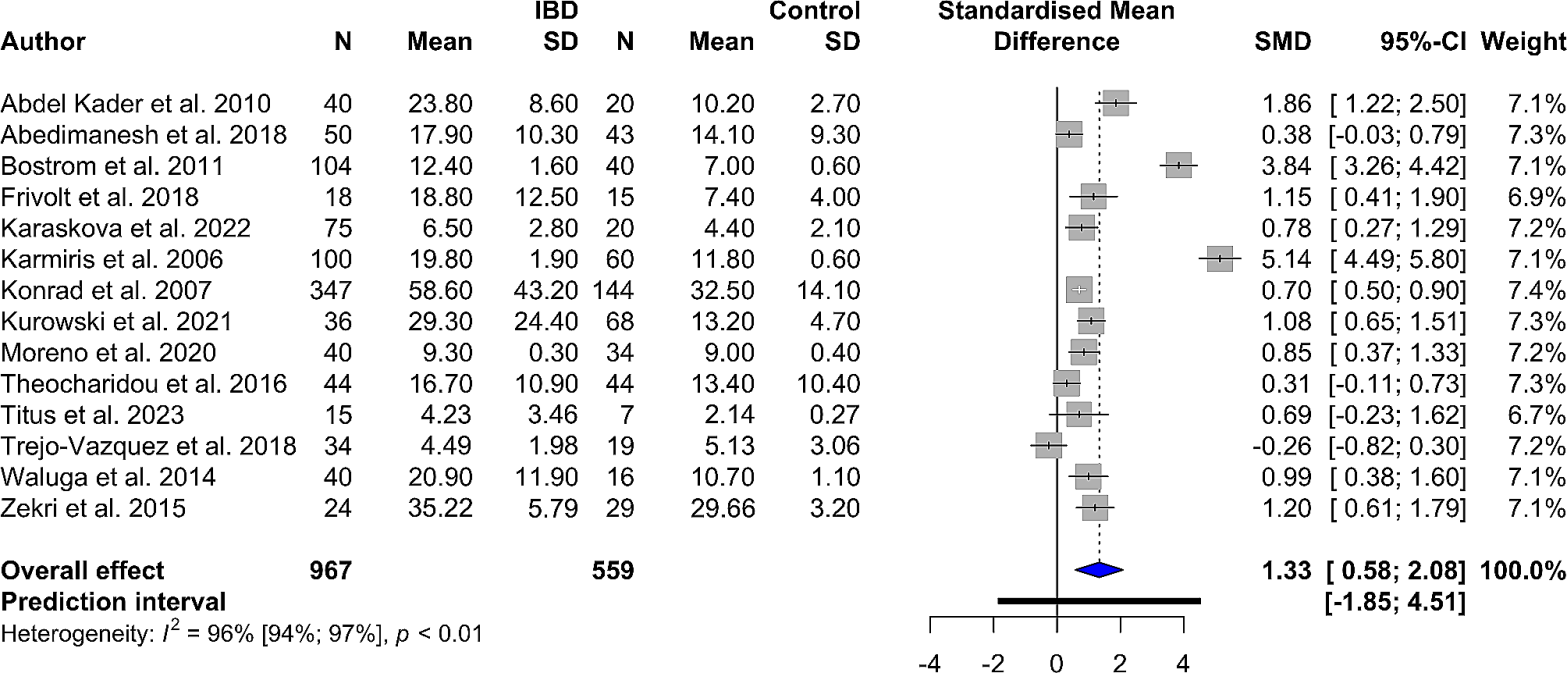
Forest plot for meta-analysis of resistin levels in patients with inflammatory bowel disease (IBD) vs. healthy controls

**Fig. 3:**
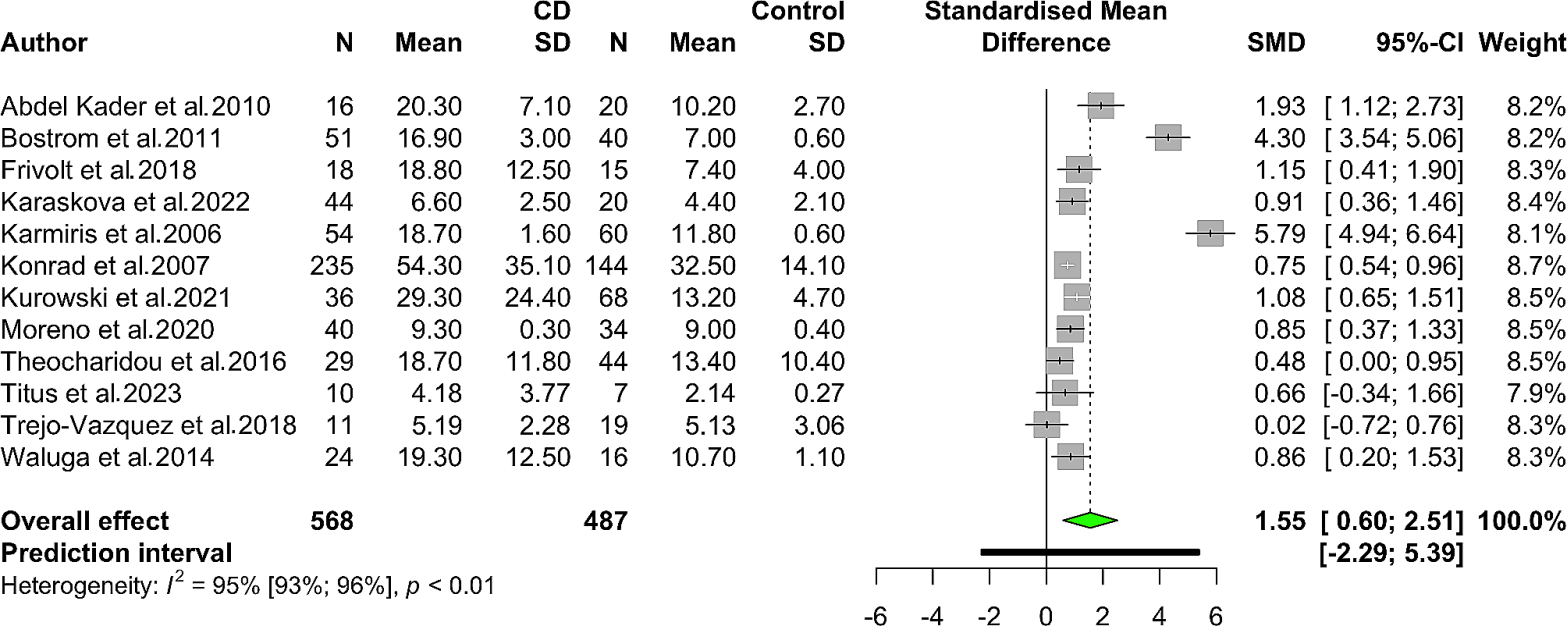
Forest plot for meta-analysis of resistin levels in patients with Crohn’s disease (CD) vs. healthy controls

**Fig. 4:**
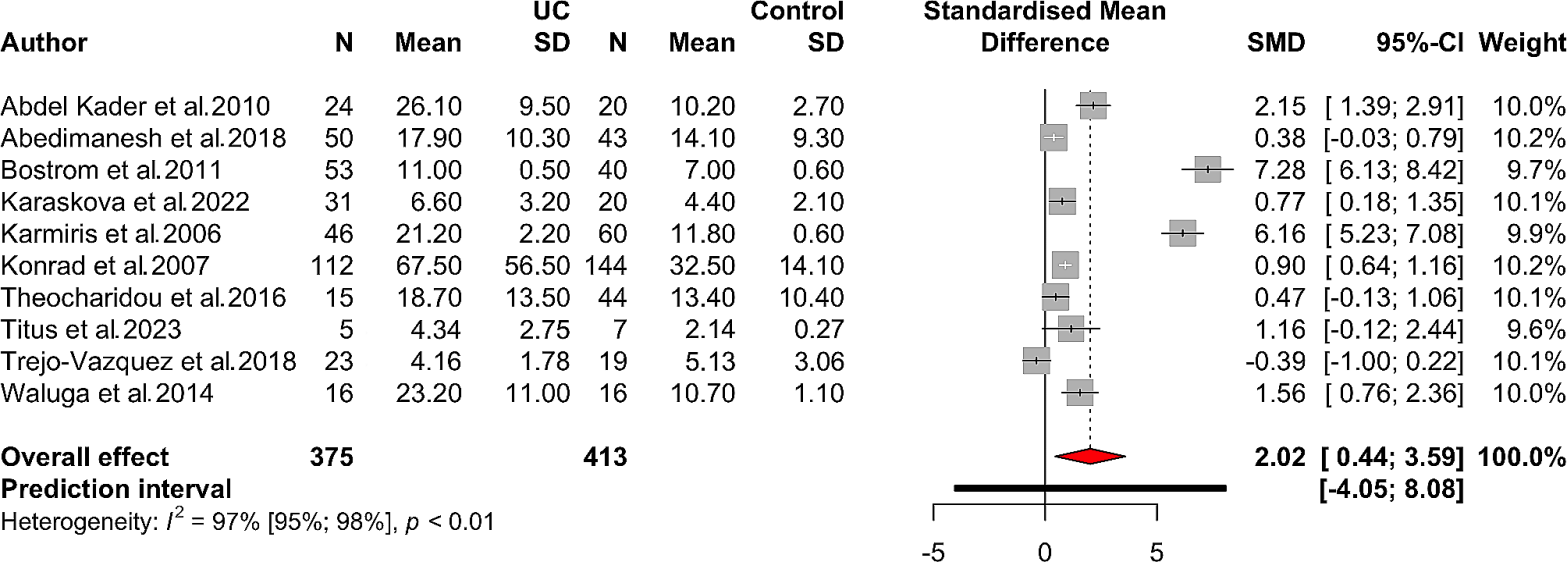
Forest plot for meta-analysis of resistin levels in patients with ulcerative colitis (UC) vs. healthy controls

Fourteen studies assessed blood resistin levels in patients with IBD compared to healthy controls. It was shown that those with IBD had significantly higher levels (SMD 1.33, 95% CI 0.58 to 2.08, *p*-value < 0.001). There was a high level of heterogeneity in this analysis (*I*^*2*^: 96%, 95% CI 94–97%). The forest plot for this meta-analysis is illustrated in Fig. 2. In a separate analysis of patients with CD, significantly higher serum/plasma resistin levels were found in CD cases (SMD 1.55, 95% CI 0.60 to 2.51, *p*-value = 0.001, *I*^*2*^: 95%) vs. controls. Higher circulating resistin levels were also found in patients with UC as compared with controls (SMD 2.02, 95% CI 0.44 to 3.59, *p*-value = 0.012, *I*^*2*^: 97%). Figures 3 and 4 represent the forest plot for meta-analyses of CD and UC, respectively.

Sensitivity analysis by the leave-one-out method is shown in Supplementary Figs. 1–3 for IBD, CD, and UC, respectively. None of the studies significantly affected the overall effect size in terms of significance after removal.

Funnel plots (counter-enhanced and trim-and-fill methods) were designed for the assessment of publication bias, as illustrated in Supplementary Figs. 4–6. There was no asymmetry in the IBD and CD funnel plots. However, the trim-and-fill funnel plot for the UC meta-analysis showed asymmetry with three added studies which resulted in an insignificant difference between patients with UC and healthy controls (SMD 0.684, 95% CI -1.245 to 2.614, *p*-value = 0.487, *I*^*2*^: 98%). Egger’s test was not significant for any of the IBD, CD, and UC analyses (*p*-value = 0.184, 0.136, and 0.109, respectively).

Due to high heterogeneity in meta-analysis, meta-regression was performed for mean age, publication year, sample size, male percentage, and mean BMI of patients with IBD for comparison of resistin levels in patients with IBD vs. healthy controls (Table 2). The only variable showing significant association with the SMD in each study was publication year, representing a slope of -0.163 (95% CI -0.283 to -0.045, *p*-value = 0.007). It also accounted for 33.3% of the variance within the studies. The bubble plots for these analyses are illustrated in Supplementary Figs. 7–11.

**Table 2:** Meta-regression analysis for meta-analysis of resistin levels in patients with IBD vs. healthy controls

Moderator	No. of Comparisons		Meta-regression				R^2^ Analog (proportion of variance explained)
	IBD	Control	Slope	95% CI		*p*-value	
**Mean Age (years)**	952	552	0.015	-0.050	0.080	0.654	0%
**Publication Year**	967	559	-0.163	-0.283	-0.045	0.007	33.3%
**Male percentage**	912	532	0.040	-0.105	0.186	0.589	0%
**Sample Size**	967	559	0.002	-0.007	0.011	0.688	0%
**Mean BMI (kg/m** ^**2**^ **)**	788	415	-0.042	-0.405	0.320	0.819	0%

### Meta-analysis of resistin levels in comparison of patients with active IBD, patients in remission, and healthy controls

**Fig. 5:**
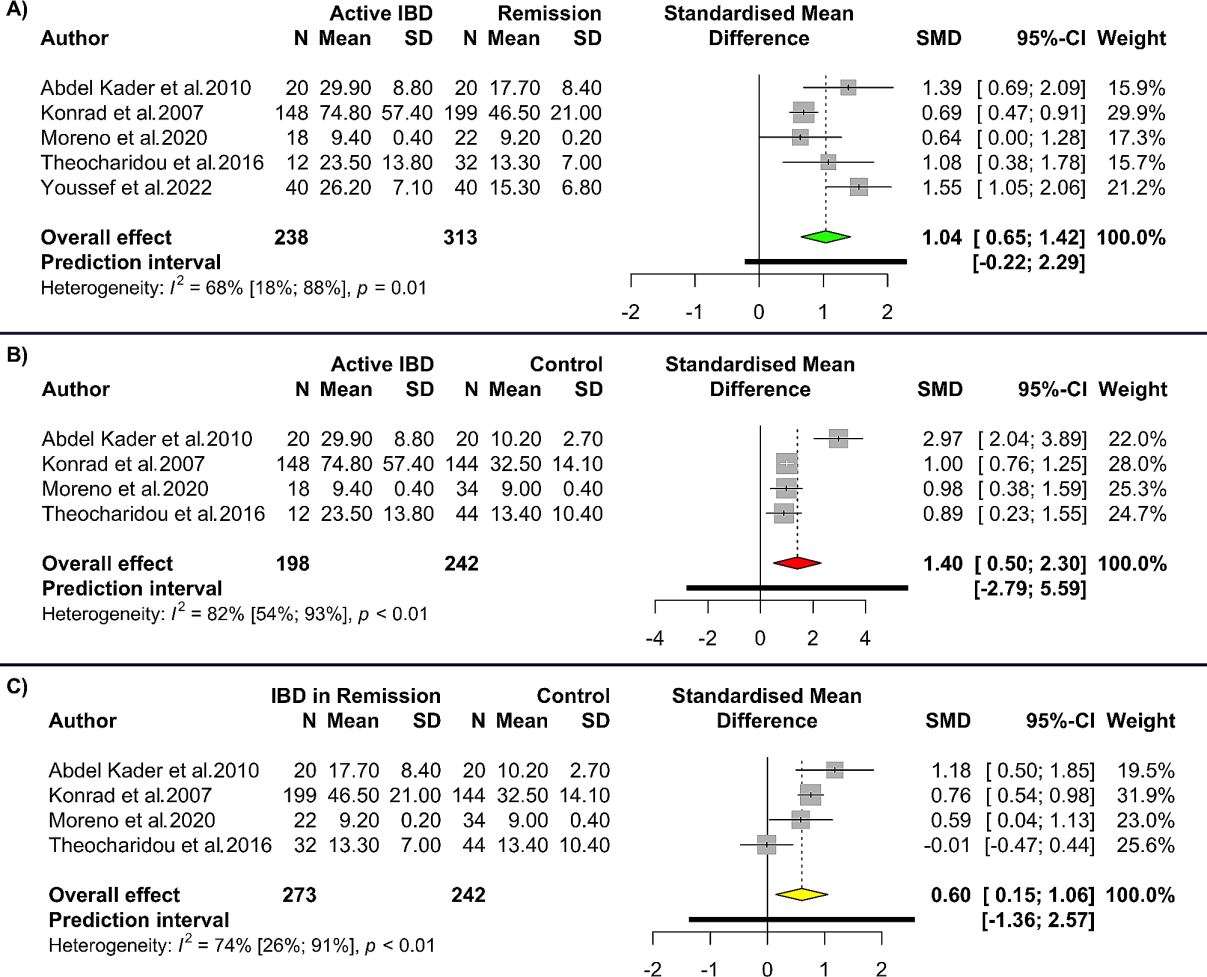
Forest plot for meta-analysis of resistin levels in (A) patients with active inflammatory bowel disease (IBD) vs. patients in remission; (B) active inflammatory bowel disease (IBD) vs. healthy controls; and (C) patients with inflammatory bowel disease (IBD) in remission vs. healthy controls

Random-effect meta-analysis revealed that patients with active IBD had significantly higher levels of resistin than those in remission (SMD 1.04, 95% CI 0.65 to 1.42, *p*-value < 0.001, Fig. 5A) which had moderate heterogeneity (*I*^*2*^: 68%). The meta-analysis of active IBD vs. healthy controls showed significantly higher levels of resistin in patients with active IBD (SMD 1.40, 95% CI 0.50 to 2.30, *p*-value = 0.002, Fig. 5B). Meta-analysis of patients in remission showed also higher levels of serum/plasma resistin in inactive IBD patients vs. healthy controls (IBD 0.60, 95% CI 0.15 to 2.57, *p*-value = 0.009, Fig. 5C).

### The discriminative ability of resistin in patients with IBD

Moreno et al. [34] was the only study that assessed discriminative ability of resistin in patients with IBD. It evaluated patients with CD vs. healthy controls and compared the discriminatory power of resistin in distinguishing (1) quiescent CD from healthy controls (AUC 0.59, 95% CI 0.43 to 0.74), (2) quiescent CD from active CD (AUC 0.66, 95% CI 0.49 to 0.83), and (3) active CD from healthy controls (AUC 0.75, 95% CI 0.61 to 0.89).

### Infliximab therapy in patients with IBD

Frivolt et al. [7] found higher serum resistin levels in patients with CD compared to healthy controls. Treatment with infliximab significantly reduced serum resistin levels in CD patients after two weeks (from 14.7 [5.1–50.5] ng/ml to 6.9 [2.9 to 16.8] ng/ml, *p*-value < 0.0001) and 14 weeks (from 14.7 [5.1–50.5] ng/ml to 9.2 [4.1–20.6] ng/ml, *p*-value = 0.0011). In line with the previous study, Karmiris et al. [20] showed that serum resistin levels were significantly decreased after treatment with infliximab (from 26.3 ± 4.1 ng/ml to 13.9 ± 1.4 ng/ml, *p*-value = 0.004). Kurowski et al. [16] found that serum resistin levels were significantly higher in CD patients who used biologic agents (93% used infliximab) compared to patients not treated with biologic agents (29.8 [12.6–57.6] ng/ml vs. 13.8 [7.8–111.1] ng/ml, *p*-value = 0.004). Resistin ≥ 29.8 ng/ml was associated with escalation to the use of biological agents (sensitivity = 53%, specificity = 95%, area under the curve (AUC) = 0.82 [0.67–0.97], *p*-value = 0.015).

## Discussion

In this study, we systematically reviewed the existing literature on the association of resistin and IBD development and activity. Our findings indicate that individuals with IBD have higher levels of resistin compared to healthy controls. Moreover, higher resistin level is found in patients suffering from active IBD compared to patients in remission. This difference in resistin levels in patients with IBD was shown to be less prominent in more recent studies, based on the meta-regression performed. This could be due to better controlling for confounding factors such as obesity and hyperlipidemia in more recent studies, however, the advancements in measuring resistin levels should also be taken into consideration.

Major types of IBD, namely UC and CD, are both characterized by remission and relapse episodes, presenting as diarrhea, rectal bleeding, abdominal pain, fatigue, and weight loss [43–45]. Although the main pathogenesis mechanism of IBD is unknown, there is evidence suggesting that a combination of genetics, environmental factors, and immunological abnormalities are important components of its etiology and development [46]. Conventional therapy in IBD patients includes amino-salicylates, corticosteroids (CSs), immunomodulators, biologics, like TNF inhibitors, and surgical resection if required. Since TNF inhibitors can induce long-term remission and alter the progression of the disease, their administration has led to a breakthrough in the treatment of IBD [47].

Pathophysiologically, CD is primarily mediated by T helper 1 (Th-1) cells, characterized by elevated levels of proinflammatory cytokines like interleukin (IL)-6 and tumor necrosis factor -alfa (TNF- α). In contrast, UC is predominantly mediated by natural killer T cells producing IL-13 or by Th2 cells producing IL-4 and IL-13 [48]. Interestingly, in CD, mucosal ulcerations tend to be particularly prominent along the mesenteric attachments, suggesting a potential link between mesenteric adipose tissue and mucosal changes [7, 18, 49]. In addition, mesenteric adipose tissue in CD patients exhibits elevated levels of TNF, C-reactive protein (CRP), and adipokines such as resistin [50]. White adipose tissue (WAT) has emerged as a major metabolic and secretory organ that participates in the production and release of several bioactive proteins, namely adipokines [51]. Adipokines are associated with the initiation and maintenance of inflammatory and immune responses [52]. Consistently, their dysregulation has been considered to play an essential role in the pathogenesis of autoimmune diseases such as IBD [53, 54]. Among various adipokines, resistin, a cysteine-rich peptide mainly produced by peripheral blood mononuclear cells (PBMC), contributes to the regulation of metabolism, adipogenesis, glucose hemostasis, and inflammatory processes [55–57].

CRP, erythrocyte sedimentation rate (ESR), anti-neutrophil cytoplasm antibodies (ANCA), anti-saccharomyces cerevisiae antibodies (ASCA), Leucine-Rich α2 Glycoprotein, and fecal calprotectin are commonly-used biomarkers as indicators of disease activity in patients with autoimmune diseases like IBD [58]. The utilization of biomarkers as diagnostic and prognostic tools in IBD offers a cost-effective, less time-consuming, and minimally invasive alternative to endoscopic procedures. Among currently used biomarkers, fecal calprotectin has been considered the gold standard for IBD diagnosis in adults [59], however, its sensitivity and specificity depend on the location of the inflammation. Also, several studies have reported lower specificity of fecal calprotectin in CD patients, rather than UC cases, and higher specificity in individuals with large bowel disease involvement, compared to small bowel disease [60–62]. As a result, many studies are focused on the identification and validation of the novel most reliable biomarkers [63]. Accordingly, previous studies have suggested that resistin may serve as a potential biomarker with high sensitivity to disease activity [33]. Additionally, recent studies revealed that resistin levels have a positive correlation with leukocyte count and CRP [64]. In the present systematic review, we observed a notable elevation in resistin levels among IBD patients compared to healthy controls. Furthermore, resistin levels remained significantly higher in patients experiencing active disease compared to those in remission. Similar results were also recognized between quiescent patients and healthy controls. These observations could be attributed to proinflammatory properties of resistin as mentioned above [28, 34]. Compared to more commonly used markers (e.g. ESR and CRP), resistin has been found to be more valuable in monitoring and better understanding the disease activity. This is primarily due to its ability to differentiate patients in recovery from healthy individuals, providing more accurate information about the disease progression [58]. Although the majority of published scholars are in line with our findings, few articles have reported controversial findings reporting a lack of significant association between resistin and IBD disease [32, 39, 42]. These studies are limited due to their patient selection and sample size. It is also important to note that obesity significantly influences circulating resistin levels due to its increased secretion by enlarged adipose tissue [65], although our meta-regression based on BMI did not show any association between resistin difference and BMI. However, it is highly suggested that the laboratory reference ranges for resistin level be standardized according to body fat mass [28]. In addition to BMI, several other factors have been shown to be associated with serum resistin levels, such as insulin resistance [66]. In the study by Norata et al., a positive correlation was found between triglycerides, waist circumference, waist/hip ratio, and systolic blood pressure, many of which are present in metabolic syndrome [67]. The association and mediator effect of these factors have not been assessed in IBD studies and hence, there is a need for further studies aiming at investigating these factors’ impact on the observed relation. They could also be the source of high heterogeneity found among the studies.

Infliximab (IFX) is an anti-TNF-α monoclonal antibody that exerts its therapeutic effect by inhibiting TNF-α associated inflammatory responses [68]. Several studies have demonstrated its safety and efficacy to induce and maintain remission in severe and complicated UC and CD patients [69, 70]. Interestingly, it was found that administration of Infliximab led to a significant reduction of resistin levels through the neutralization of TNF-α [7, 20]. This observation suggests that resistin may be a good marker to evaluate the response to treatment with IFX. Moreover, this observation raises the question if resistin could be used as a target in treating patients with IBD. As of today, no study has evaluated targeting resistin as a therapeutic method in IBD. Future studies evaluating novel drugs and biological agents targeting resistin are required to clarify if such treatments have a role in the therapeutic approach toward IBDs.

Our findings have several clinical implications. Our study demonstrated that serum/plasma resistin levels were higher in patients with IBDs compared with controls. This can emphasize the diagnostic utility of this adipocyte as a reliable easy-to-measure noninvasive biomarker. Since our analyses had high heterogeneity that could stem from different methodologies, different populations, and different clinical settings, further diagnostic studies aiming at determining its sensitivity and specificity in different regions and among different IBD populations can lead to its clinical use. Similarly, since resistin was significantly higher in patients with active disease, its measurement could lead to better disease activity monitoring. However, before that, there is a need for further studies aiming at assessing the discriminative ability of resistin by assessing sensitivity, specificity, and AUC since the only study that reported this found an AUC of 0.66 for this which is far from ideal. Given the fact that this was only one study and the meta-analysis found a significant difference between active cases and those in remission, further studies are warranted to address this issue.

This systematic review and meta-analysis has several strengths. We performed a comprehensive and rigorous systematic search methodology, ensuring that all relevant studies were included in the analysis. This approach enhances the reliability of our investigations. Also, we conducted a quality assessment of the included studies using the Newcastle-Ottawa quality rating scale. Therefore, all included studies were highly qualified. However, we should acknowledge several limitations of our study. First, the small size of the studied population and sample selection bias could be a limitation that might be attributed to the study design in which variations of potential confounders were not considered. Second, among the included studies, there is no direct comparison of currently used biomarkers and resistin which highlights the need for further studies comparing these. Moreover, the methods for measuring resistin levels were not the same among the studies. Although we used SMD rather than MD in order to minimize this effect, this could be a limitation of our findings. Fourth, regarding the diagnostic ability of resistin for IBD, only one study performed ROC-AUC analysis and hence, we were unable to perform meta-analysis in this regard. Future studies should highly consider the calculation of sensitivity, specificity, and AUC for this biomarker. Fifth, as resistin is affected by several conditions [67], the differences in populations of included studies might limit our findings. Finally, the high heterogeneity observed in most of our analyses was not resolved through meta-regression, except for publication year which accounted for 33% of the variance. This could stem from varied clinical settings, populations, and assessment methods among studies. Wherever possible, we separated UC and CD cases in order to homogenize the studies which could not lower the heterogeneity.

## Conclusions

In this systematic review, we demonstrated that blood resistin level has an association with IBD development and activity. It could have clinical implications since resistin can be measured in peripheral blood and it might become an ideal candidate for monitoring disease activity. The association was also significant in IBD patients on IFX therapy. However, it is important to note that current findings are inconsistent, and further studies with larger populations and a more standardized methodology are necessary to obtain conclusive results.

### Electronic supplementary material

Below is the link to the electronic supplementary material.

Supplementary Material 1

## Data Availability

The datasets used and/or analyzed during the current study are available from the corresponding author upon reasonable request.
