# Supplementary material for: Circulatory resistin levels in inflammatory bowel disease: a systematic review and meta-analysis
Source: BMC Gastroenterol. 2024 Mar 14;24:107. doi: 10.1186/s12876-024-03199-7 (PMC10941394; doi:10.1186/s12876-024-03199-7)
Supplement: Supplementary file 1 — Supplementary Material 1 [file 12876_2024_3199_MOESM1_ESM.docx]

**Supplementary Materials**

***Supplementary Table 1.*** *The search queries used for each database and the search results*

| **Query** | | **Results (No.)**  **(7 June 2023)** |
| --- | --- | --- |
| **PubMed** | | |
| #1 | (“inflammatory bowel disease*”[tiab] OR “IBD”[tiab] OR “Crohn*”[tiab] OR “Crohn's disease”[tiab] OR “ulcerative colitis”[tiab] OR “UC”[tiab] OR “CD”[tiab] OR "Inflammatory Bowel Diseases"[Mesh] OR "Crohn Disease"[Mesh] OR "Colitis, Ulcerative"[Mesh]) | 296,126 |
| #2 | (“resistin”[tiab] OR " FIZZ3”[tiab] OR “RETN”[tiab] OR "Resistin"[Mesh] OR “RELM”[tiab] OR "adipose tissue-specific secretory factor definition " [tiab] OR “Adipocyte-specific secretory factor”[tiab] OR “ADSF”[tiab] OR “resistin-like molecule”[tiab]) | 5,328 |
| #3 | #1 AND #2 | **71** |
| **Embase** | | |
| #1 | (“inflammatory bowel disease*” OR “IBD” OR “Crohn*” OR “ulcerative colitis” OR “UC” OR “CD”) | 540,623 |
| #2 | (“resistin” OR " FIZZ3” OR “RETN” OR “RETN” OR “RELM” OR "adipose tissue-specific secretory factor definition" OR “Adipocyte-specific secretory factor” OR “ADSF” OR “resistin-like molecule”) | 10,501 |
| #3 | #1 AND #2 | **249** |
| **Web of Science** | | |
| #1 | TS=(“inflammatory bowel disease*” OR “IBD” OR “Crohn*” OR “ulcerative colitis” OR “UC” OR “CD”) | 473,961 |
| #2 | TS=(“resistin” OR " FIZZ3” OR “RETN” OR “RETN” OR “RELM” OR "adipose tissue-specific secretory factor definition" OR “Adipocyte-specific secretory factor” OR “ADSF” OR “resistin-like molecule”) | 6,391 |
| #3 | #1 AND #2 | **110** |
| **Scopus** | | |
| #1 | TITLE-ABS-KEY(“inflammatory bowel disease*” OR “IBD” OR “Crohn*” OR “ulcerative colitis” OR “UC” OR “CD”) | 622,121 |
| #2 | TITLE-ABS-KEY(“resistin” OR " FIZZ3” OR “RETN” OR “RETN” OR “RELM” OR "adipose tissue-specific secretory factor definition" OR “Adipocyte-specific secretory factor” OR “ADSF” OR “resistin-like molecule”) | 8,691 |
| #3 | #1 AND #2 | **188** |
| **Total records** | | **618** |
| **Total records after removing duplicates** | | **351** |

***Supplementary Table 2****. Quality Assessment of Included Studies Based on Newcastle-Ottawa Scale (NOS)*

| **Study** | **Selection** | | | | **Comparability** | **Outcome** | | **Overall**  **Score** |
| --- | --- | --- | --- | --- | --- | --- | --- | --- |
|  | **Representation** | **Sample size** | **Non-Respondents** | **Exposure** |  | **Outcome** | **Statistical test** |  |
| **Abdel Kedar et al. 2010** | * | * | * | * | ** | ** | * | 10 |
| **Abedimanesh et al. 2018** | * | * | * | * | ** | ** | * | 10 |
| **﻿Bostrom et al. 2011** | * | * | * | * | - | ** | * | 8 |
| **Frivolt et al. 2018** | * | * | * | * | ** | ** | * | 10 |
| **Karaskova et al. 2022** | * | * | * | * | ** | ** | * | 10 |
| **Karmiris et al 2006** | * | * | * | * | ** | ** | * | 10 |
| **Karmiris et al. 2007** | * | * | * | * | - | ** | * | 8 |
| **Konrad et al. 2007** | * | * | * | * | - | ** | * | 8 |
| **Kurowski et al. 2021** | * | * | * | * | - | ** | * | 8 |
| **Moreno et al. 2020** | * | * | * | * | - | ** | * | 8 |
| **Morshedzadeh et al. 2023** | * | * | * | * | - | ** | * | 8 |
| **﻿Sobolewska-Włodarczyk et al. 2020** | * | * | * | * | - | ** | * | 8 |
| **Theocharidou et al. 2016** | * | * | * | * | ** | ** | * | 10 |
| **Titus et al. 2023** | * | * | * | * | - | ** | * | 8 |
| **Trejo-Vazquez et al. 2018** | * | * | * | * | - | ** | * | 8 |
| **Valentini et al. 2009** | * | * | * | * | ** | ** | * | 10 |
| **Waluga et al. 2014** | * | * | * | * | ** | ** | * | 10 |
| **Youssef et al. 2022** | * | * | * | * | - | ** | * | 8 |
| **Zekri et al. 2015** | * | * | * | * | - | ** | * | 8 |

*
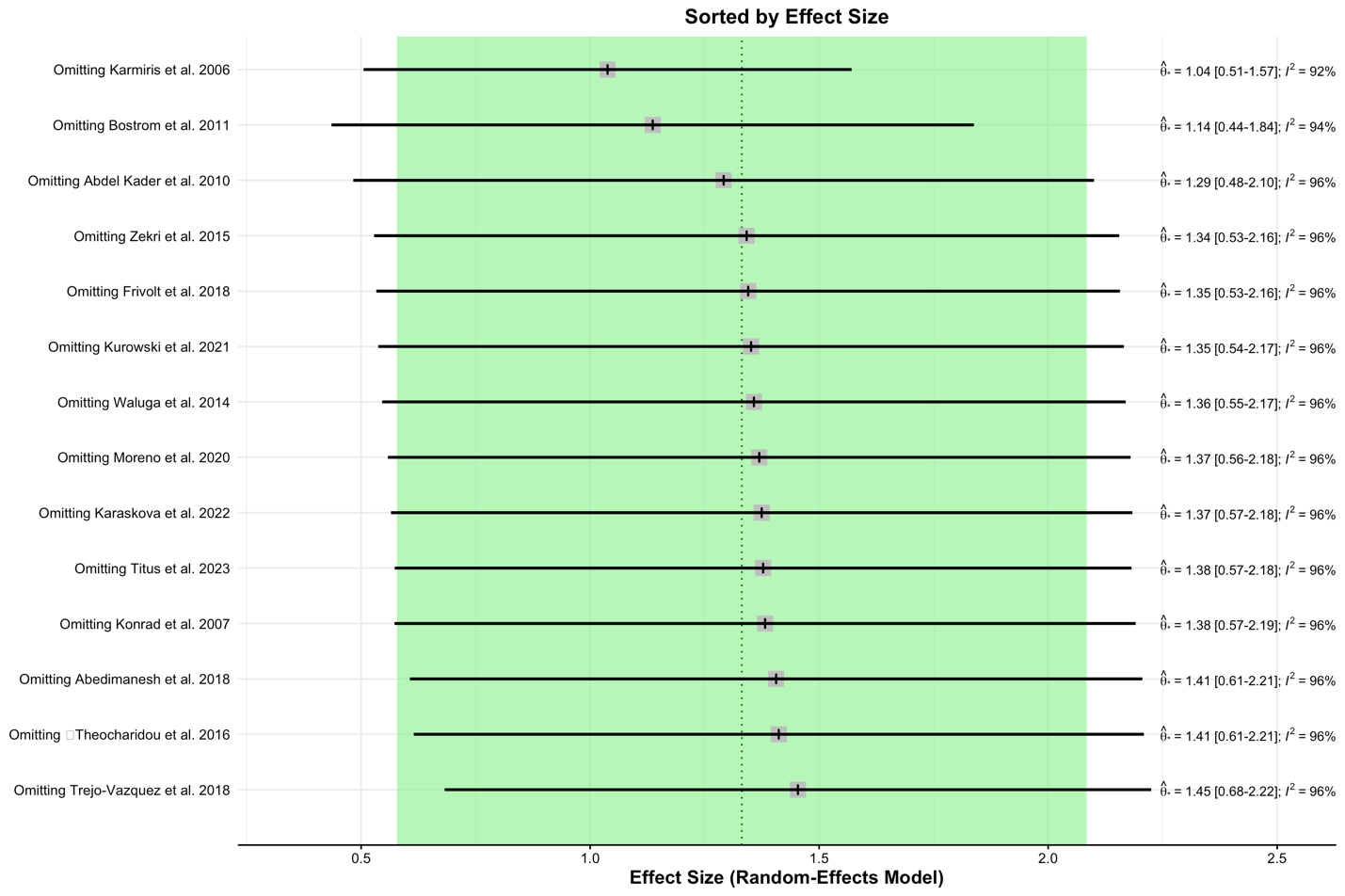
*

***Supplementary Figure 1.*** *Sensitivity analysis by leave-one-out method for meta-analysis of resistin levels in patients with IBD vs. healthy controls*

*
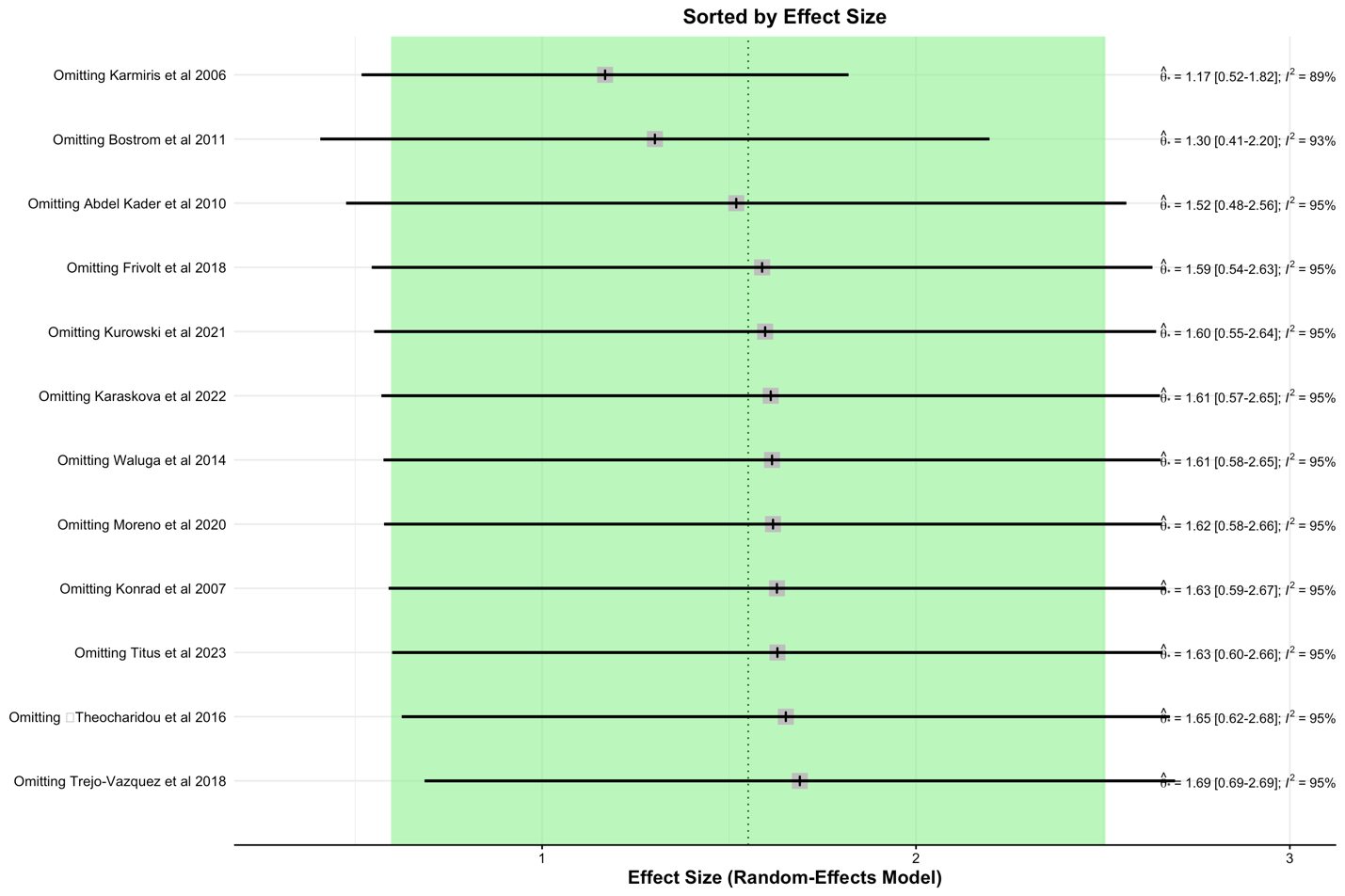
*

***Supplementary Figure 2.*** *Sensitivity analysis by leave-one-out method for meta-analysis of resistin levels in patients with CD vs. healthy controls*

*
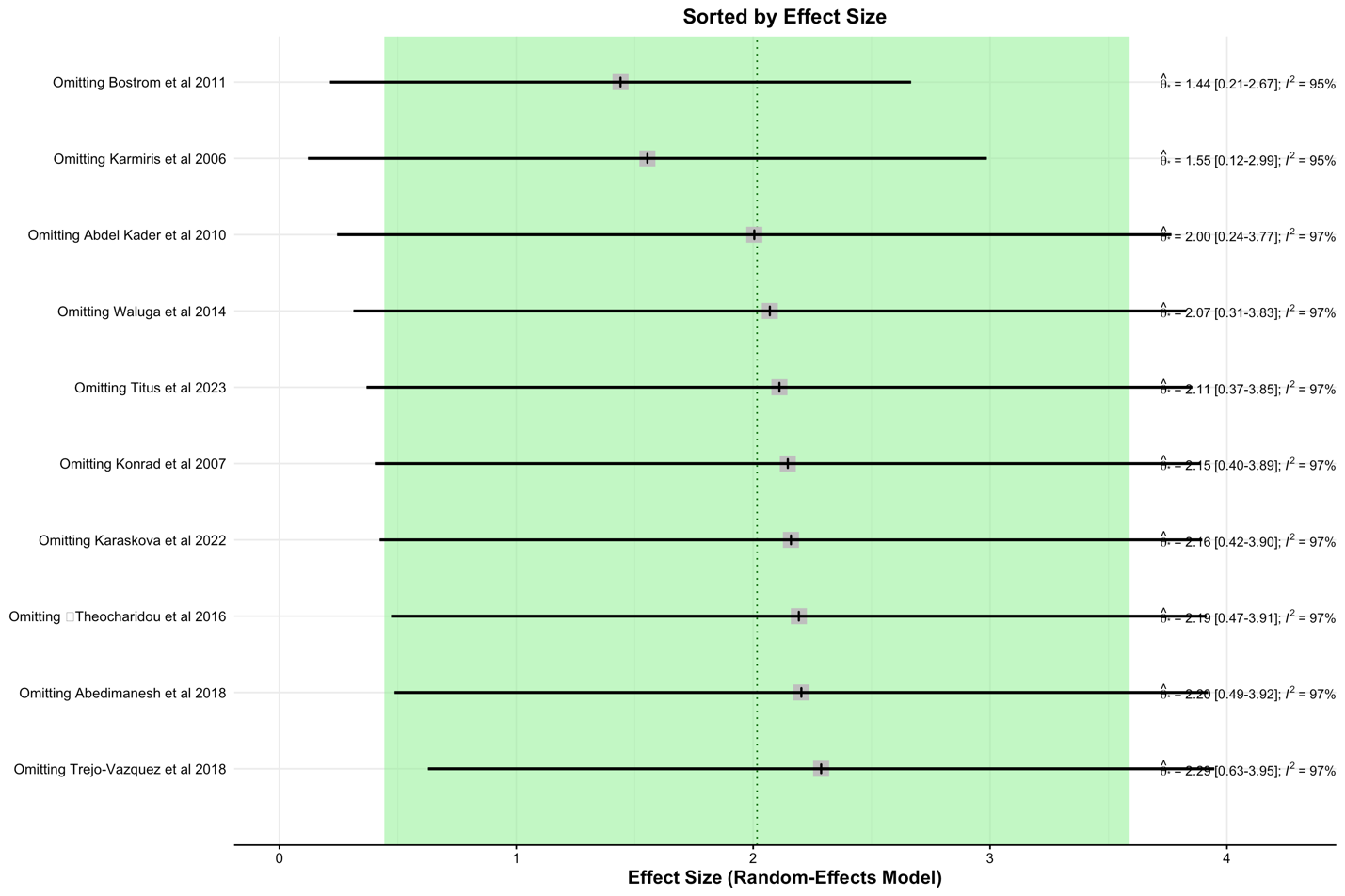
*

***Supplementary Figure 3.*** *Sensitivity analysis by leave-one-out method for meta-analysis of resistin levels in patients with UC vs. healthy controls*

*
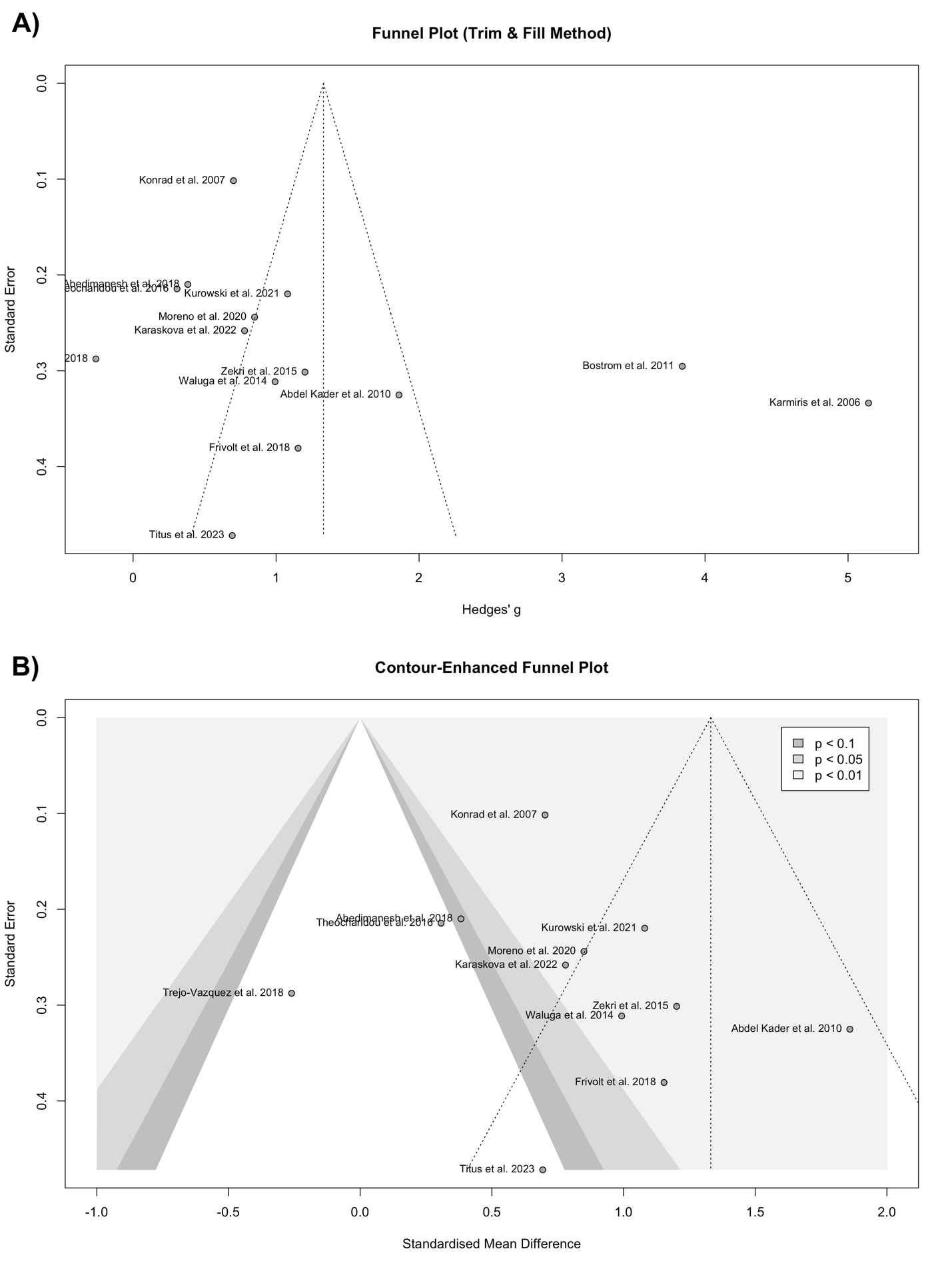
*

***Supplementary Figure 4.*** *Funnel plots for meta-analysis of resistin levels in patients with IBD vs. healthy controls; A) Trim and Fill method and B) Countor-enhanced funnel plot*

*
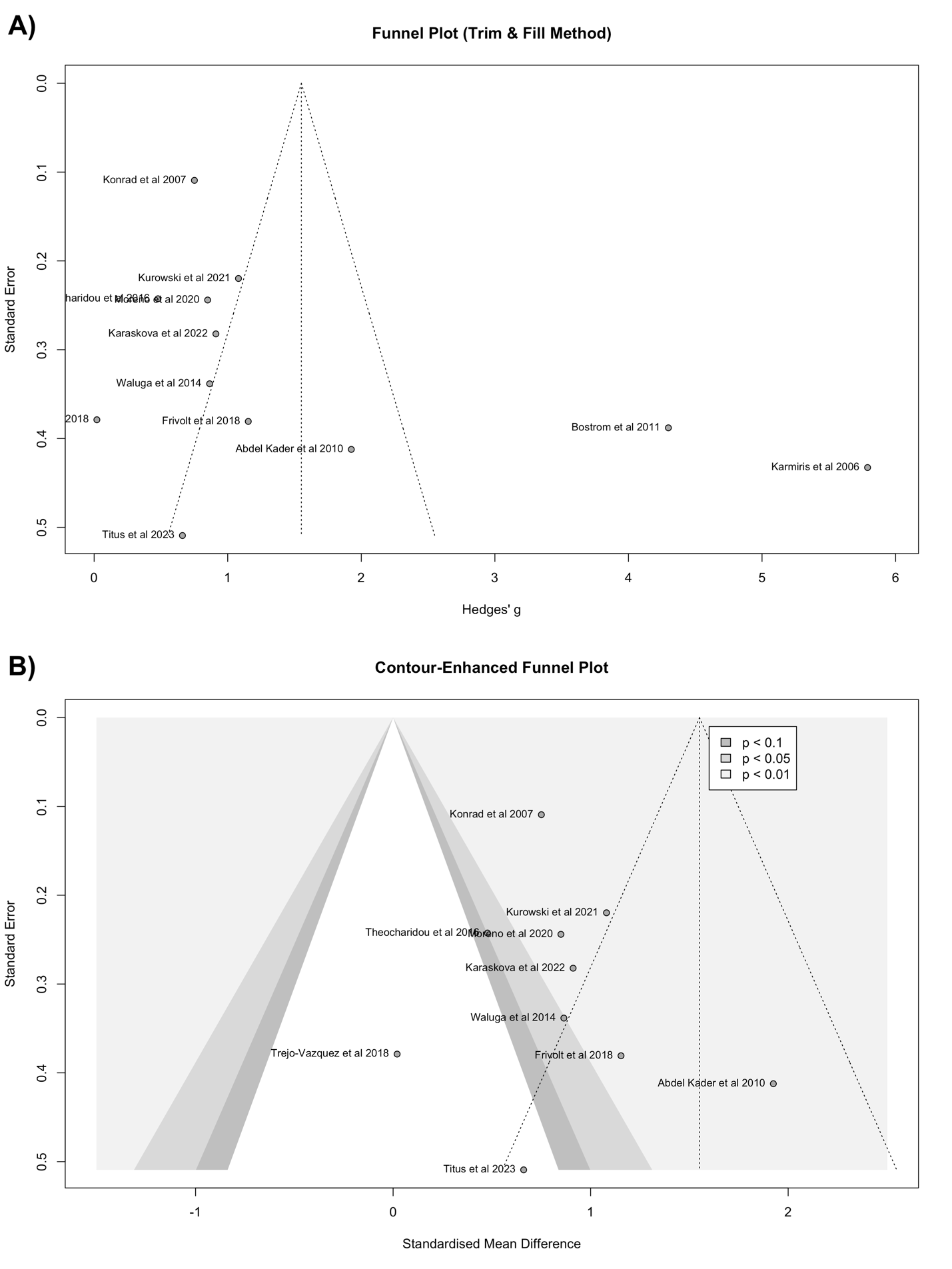
*

***Supplementary Figure 5.*** *Funnel plots for meta-analysis of resistin levels in patients with CD vs. healthy controls; A) Trim and Fill method and B) Countor-enhanced funnel plot*

*
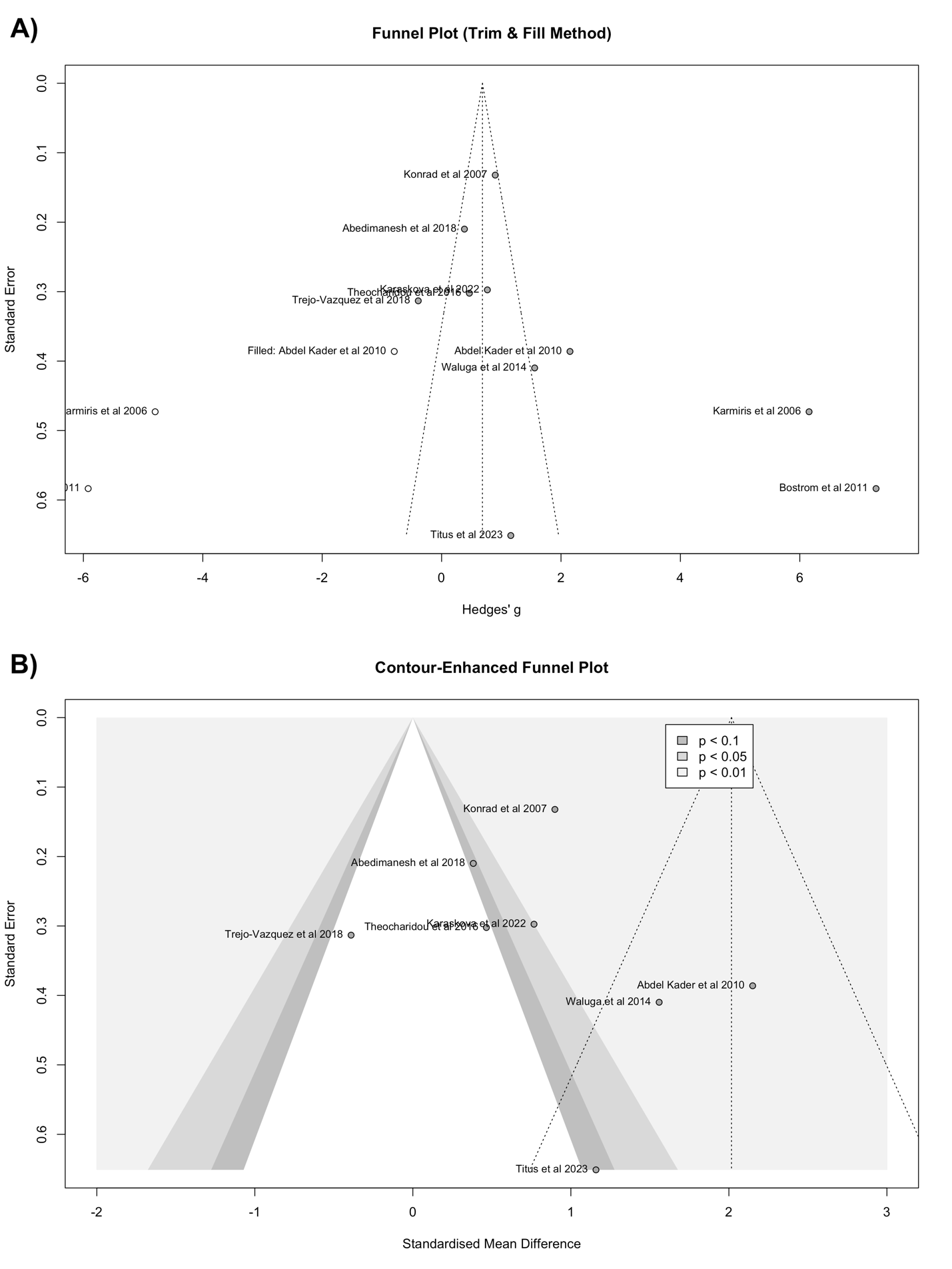
*

***Supplementary Figure 6.*** *Funnel plots for meta-analysis of resistin levels in patients with UC vs. healthy controls; A) Trim and Fill method and B) Countor-enhanced funnel plot*

***
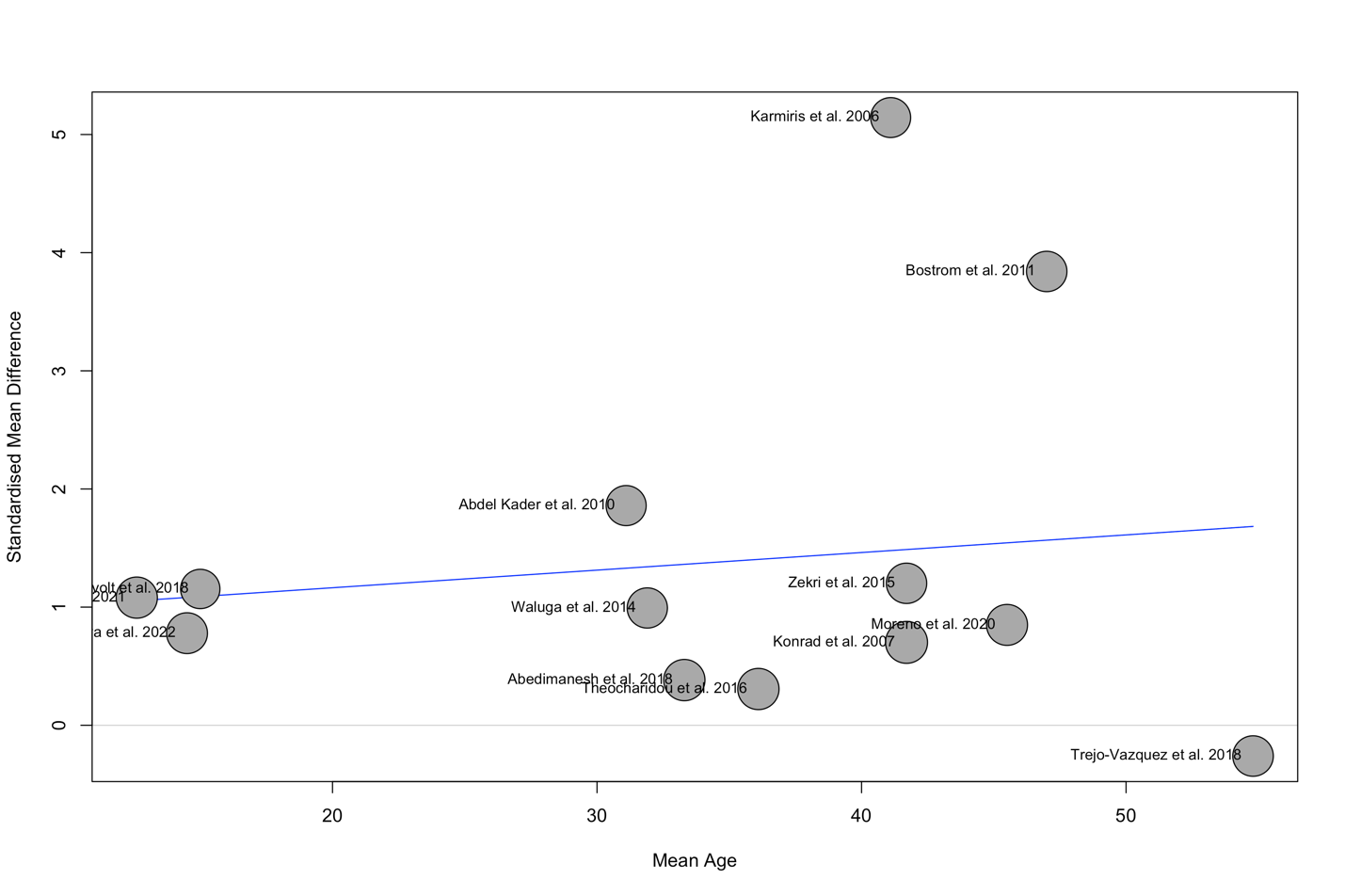
***

***Supplementary Figure 7.*** *Bubble plot showing the meta-regression of mean age for meta-analysis of resistin levels in patients with IBD vs. healthy controls*

***
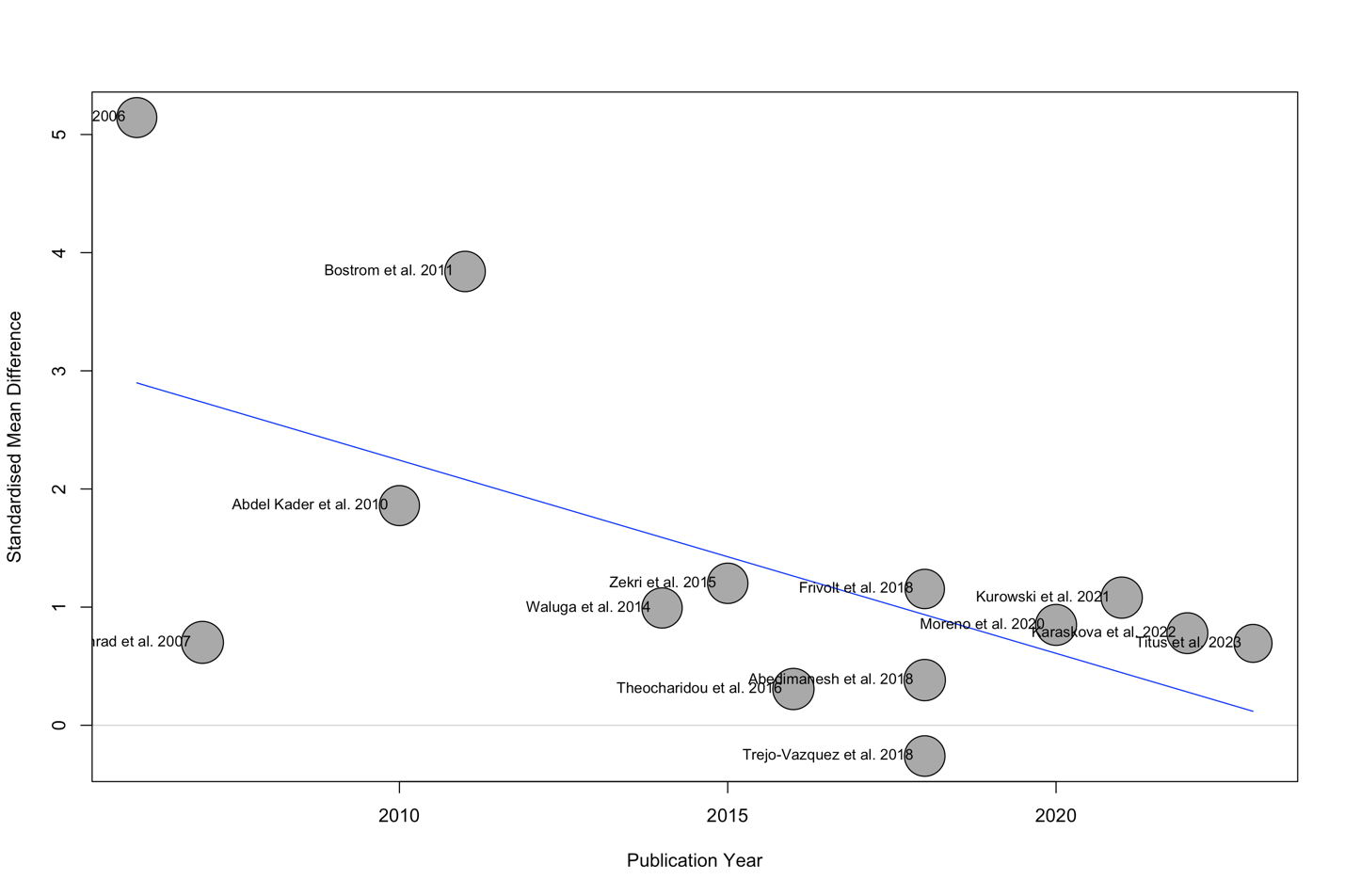
***

***Supplementary Figure 8.*** *Bubble plot showing the meta-regression of publication year for meta-analysis of resistin levels in patients with IBD vs. healthy controls*

***
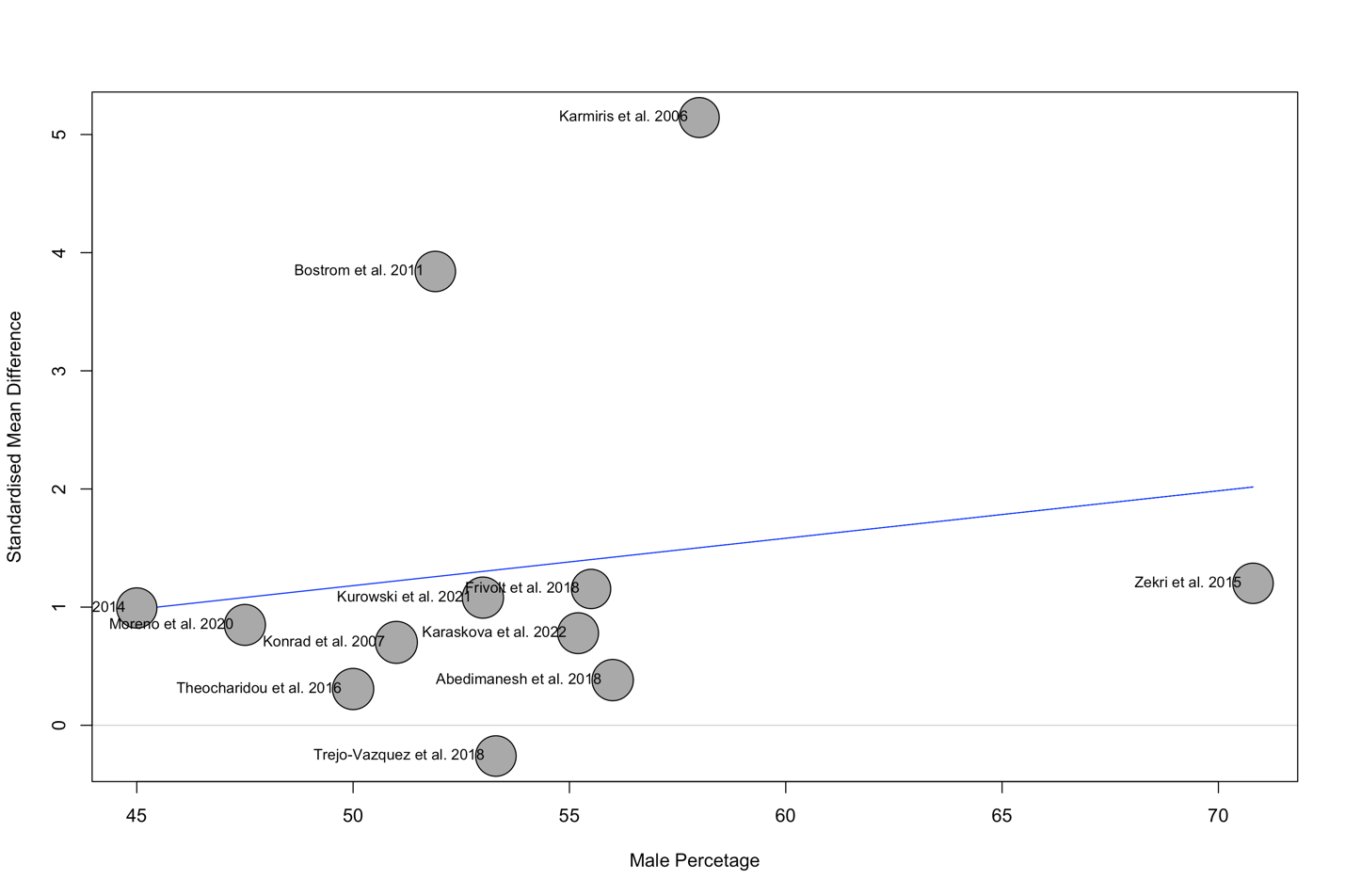
***

***Supplementary Figure 9.*** *Bubble plot showing the meta-regression of male percentage for meta-analysis of resistin levels in patients with IBD vs. healthy controls*

***
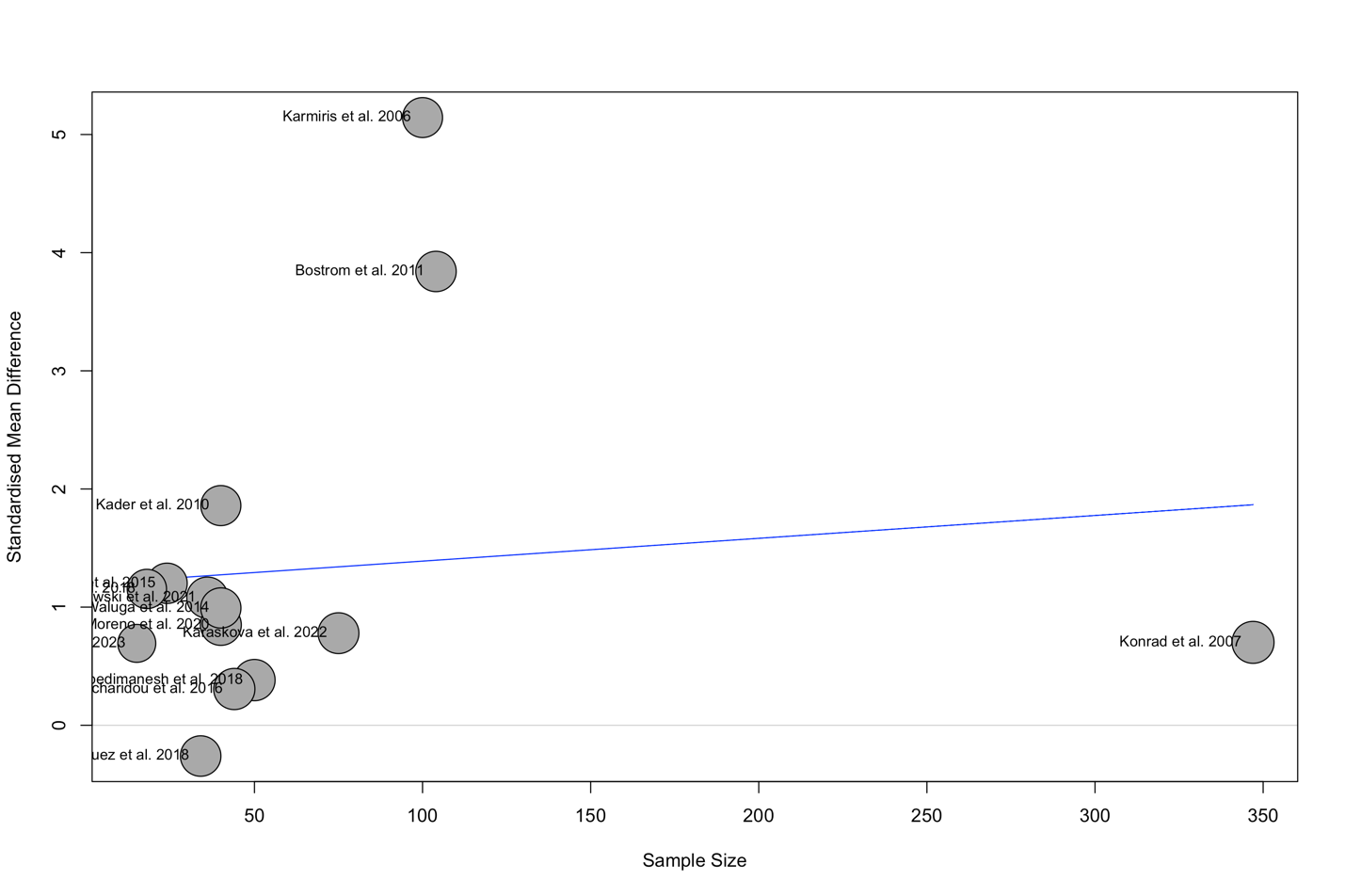
***

***Supplementary Figure 10.*** *Bubble plot showing the meta-regression of sample size for meta-analysis of resistin levels in patients with IBD vs. healthy controls*

***
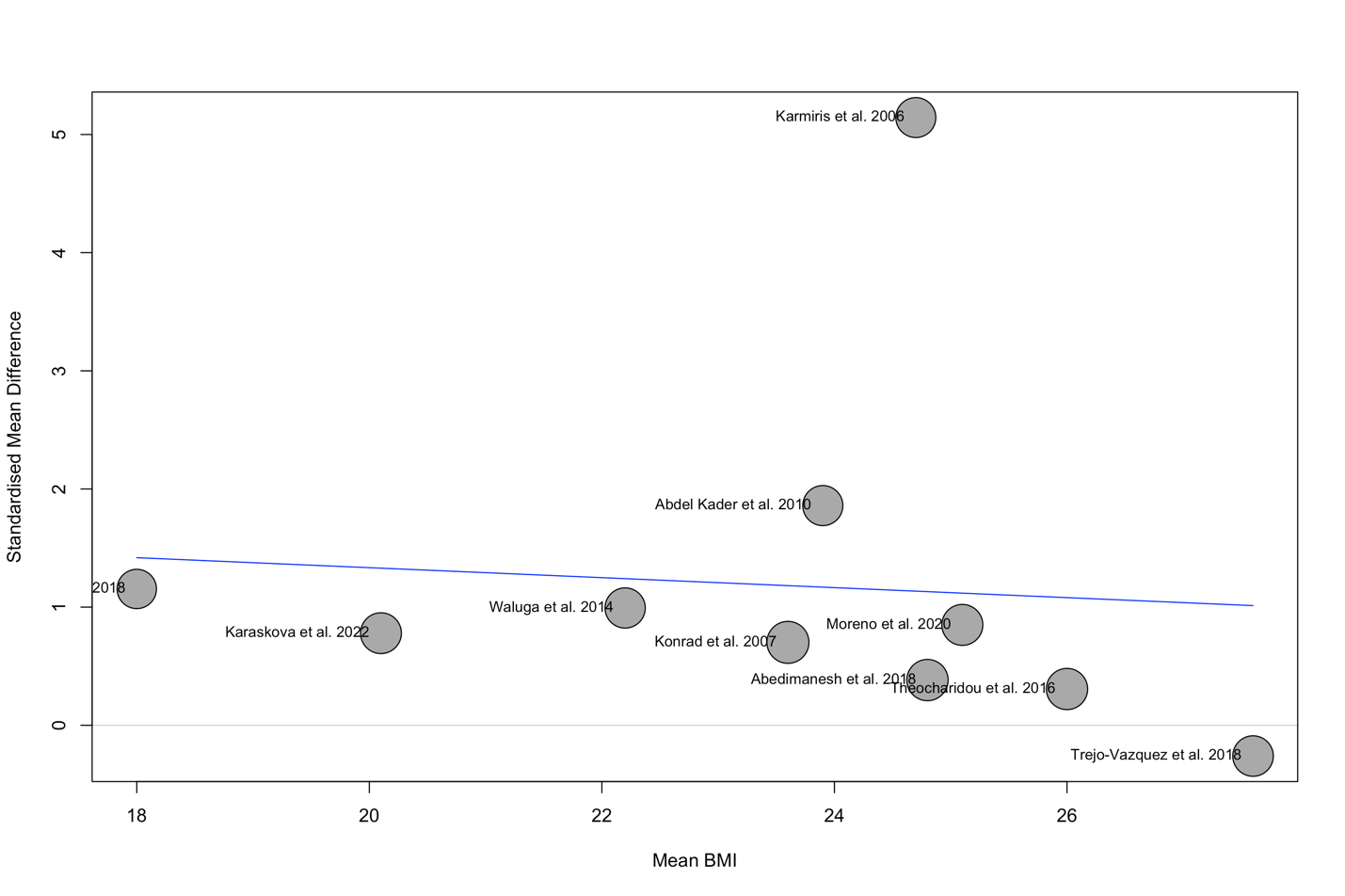
***

***Supplementary Figure 11.*** *Bubble plot showing the meta-regression of mean BMI for meta-analysis of resistin levels in patients with IBD vs. healthy controls*
